# Discrepancies between Abstracts Presented at International Association for Dental Research Annual Sessions from 2004 to 2005 and Full-Text Publication

**DOI:** 10.1155/2012/859561

**Published:** 2012-02-22

**Authors:** Soni Prasad, Damian J. Lee, Judy Chia-Chun Yuan, Valentim A. R. Barao, Nodesh Shyamsunder, Cortino Sukotjo

**Affiliations:** ^1^Department of General Dental Sciences, Marquette University School of Dentistry, 1801 W Wisconsin Avenue, Milwaukee, WI 53233-2186, USA; ^2^Department of Restorative Dentistry, College of Dentistry (MC 555), University of Illinois at Chicago, 801 South Paulina Street, Room 365B, Chicago, IL 60612-7211, USA; ^3^Department of Dental Materials and Prosthodontics, Aracatuba Dental School, Jose Bonifacio, 1193, 16015-050 Aracatuba, SP, Brazil; ^4^Advanced Education in Prosthodontics, College of Dental Medicine, Nova Southeastern University, 3301 College Avenue, Fort Lauderdale, Davie, FL 33314-7796, USA

## Abstract

*Purpose*. The purpose of this study was to evaluate the discrepancies between abstracts presented at the IADR meeting (2004-2005) and their full-text publication. *Material and Methods*. Abstracts from the Prosthodontic Section of IADR meeting were obtained. The following information was collected: abstract title, number of authors, study design, statistical analysis, outcome, and funding source. PubMed was used to identify the full-text publication of the abstracts. The discrepancies between the abstract and the full-text publication were examined, categorized as major and minor discrepancies, and quantified. The data were collected and analyzed using descriptive analysis. Frequency and percentage of major and minor discrepancies were calculated. *Results*. A total of 109 (95.6%) articles showed changes from their abstracts. Seventy-four (65.0%) and 105 (92.0%) publications had at least one major and one minor discrepancies, respectively. Minor discrepancies were more prevalent (92.0%) than major discrepancies (65.0%). The most common minor discrepancy was observed in the title (80.7%), and most common major discrepancies were seen in results (48.2%). *Conclusion*. Minor discrepancies were more prevalent than major discrepancies. The data presented in this study may be useful to establish a more comprehensive structured abstract requirement for future meetings.

## 1. Introduction

The International Association for Dental Research (IADR) is an important venue for presentation of new research. The abstracts that are presented here often impact the attendees and are cited in many lectures and textbooks [1]. In dentistry, clinical relevant data from abstracts may find its way into day-to-day clinical practice. Any application of ideas, whether research or clinical, should be evidence based, and only full-text articles published in peer-reviewed journals are considered as the gold standard of evidence-based dentistry [2]. The data presented in abstracts are often preliminary results, and many of the abstracts do not get published in any peer-reviewed journals. Abstracts not only offer limited information [1, 3], but are also inadequate in conveying and interpreting data [4]. Therefore, it is imperative that the attendees understand the limitations in using the data derived from the abstracts [5]. For this reason, many peer-reviewed journals discourage referencing abstracts from scientific meetings [6]. 

Several groups have investigated the publication rate of abstracts presented at different health professional meetings, and it ranged from 10% to 78% [4, 7–21]. Corry reported the publication rate of ten percent randomly selected IADR/AADR abstracts from 21.6% to 24.0% in 1983 and 1984, respectively. Other studies have further examined the reasons why the abstracts do not get published [20–24]. The barriers to subsequent publications of abstracts were most often found to be because of the fact that 47% of the investigators did not have time to prepare a manuscript for publication, whereas 31% stated that the research was still ongoing [22]. Therefore, nonpublication of abstracts was usually due to failure to submit the paper rather than rejection from a journal [22]. Other reported reasons for failure to publish include poor quality of research design, small sample size, and negative findings [4]. 

It is suggested that delay in publication increases the likelihood of discrepancies between abstracts and its full-text publication [16]. Delays in publication are most often due to failure of the authors to submit the paper to a peer-reviewed journal in a timely fashion or due to an extended revision time during the review process [9, 25]. It is interesting to note that sometimes abstracts may present preliminary results that show a significant finding but fail to present a significant finding in the full-text publication [25].

Several studies have reported on the discrepancies between abstracts and full-text publications [2, 4, 26]. Some studies have reported discrepancies between abstracts and full-text publication in the number of authors and title of the publication [16, 21]. The reason in changes in the title could be due to the strict word limit in the abstract guidelines. Randomized controlled trials were found to have fewer changes in the authorship, perhaps because these studies are better planned, and the roles of each individual were made clear from the beginning [16]. Discrepancies were also observed in the study design between abstract and full-text publication. Narine et al. performed a systematic review of all abstracts published in a medical journal in 1989 and found that more than 50% inadequately described the study design and methodology [27].

In spite of being well documented in the medical literature, there is a limited knowledge on the accuracy of the data presented and interpreted in the abstract as compared to their full-text publication in dentistry. Our previous study examined the demographics of abstracts, and other data of abstracts presented at the IADR meeting (2004 to 2005) [28]. The purpose of this study was to evaluate the discrepancies between abstracts presented at the Prosthodontic section of IADR meeting (from 2004 to 2005) and their full-text publication, with regards to the title, authorship, purpose of study, materials and methods, sample size, statistical analysis, funding, result, and conclusion. Suggestions have also been made to improve the quality of abstracts submitted at the meeting, so that there is minimum discrepancy between them and their full-text publication.

## 2. Materials and Methods

**Figure 1:**
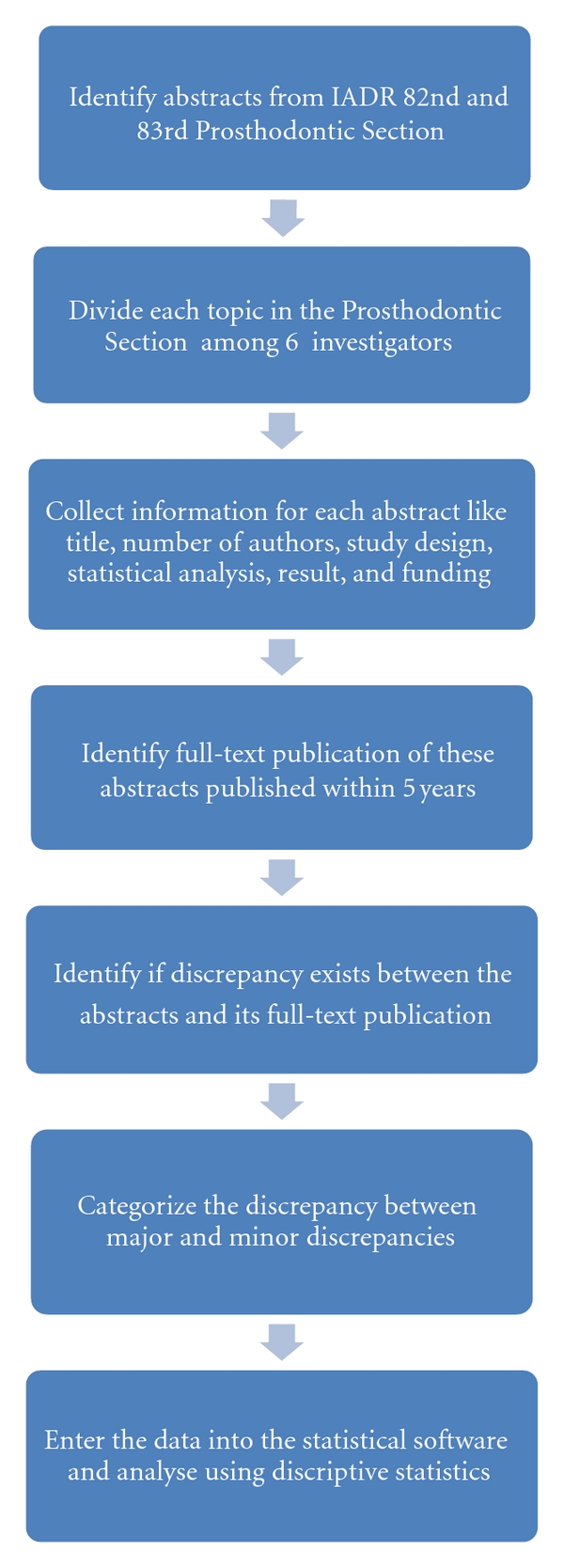
Flow chart of materials and methods.

Abstracts from poster and oral presentation for Prosthodontic Research Section of IADR 82nd and 83rd General Sessions (March 2004 and 2005) were obtained and divided among the six investigators (Figure 1). The investigators extracted data independently. For each abstract, the following basic information was collected: abstract topic, type of abstract, number of authors, study design, material and methods, statistical analysis, result, conclusion, study outcome, and funding source. To identify the full-text publication of an abstract in a peer-reviewed journal, an electronic database search was performed, using PubMed (http://www.ncbi.nlm.nih.gov/ or http://www.pubmed.gov/). Boolean operator (OR) that included all manuscripts by the first, second, and last authors was performed [15]. In cases where multiple publications were found, Boolean operator (AND) was used to combine author names and keywords from the abstract title to obtain the correct manuscript [4]. All articles with a publication date prior to the IADR meeting, published articles without access, or articles published in non-English language were excluded from the study. 

The discrepancies between the abstract presented and the full-text publication were examined and compared. These included changes in title, authorship (number of authors, the name of first and last authors), purpose, study design, materials and methods, sample size, statistical analysis, result, conclusion, study outcome, and sources of funding. The classification of each independent variable was adapted from previous publications [2, 16, 29]. Changes in sample size were recorded, and the extents of differences were noted. If the sample size was not mentioned in the abstract, it was documented as unknown. 

The discrepancies between the abstract and the full-text manuscript were categorized as major and minor discrepancies and subsequently quantified [2]. Major discrepancies consisted of changes in study purpose, materials and methods, sample size, statistical analysis, study outcome, result, and conclusion. Minor discrepancies included changes in title (such as deletion of a pronoun and use of punctuation) [29], number of authors, name of first and last authors, and sources of funding. 

To ensure data reliability among the six investigators, calibration meetings were held regularly to assess if all determinations coincided. Whenever there was a conflict or uncertainty, final group decisions were made. If a decision could not be made, group discussion followed by consensus was conducted. 

**Figure 2:**
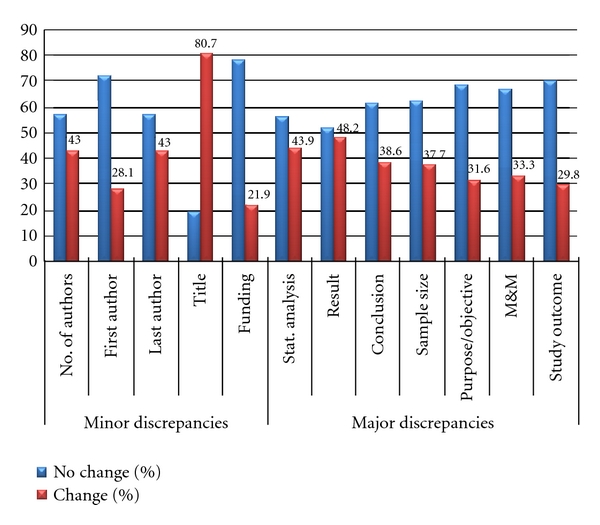
Comparison between minor and major discrepancies.

Data was entered into a software database (Microsoft Excel 2003; Microsoft, Seattle, WA), and statistical software (Statistical Package for the Social Sciences, version 17.0; SPSS Inc, Chicago, Ill) was used for the statistical analysis. Data was analyzed using descriptive statistics. Frequency and percentage were used for description of demographic data. Major and minor discrepancies between the abstract and its respective full-text publication were calculated (Figure 2). 

## 3. Results

Overall, 371 abstracts were submitted for the Prosthodontic Section of the IADR meeting between the years 2004 and 2005, and a total of 128 articles were published within a 5-year follow-up period. Out of the 128 published articles, 14 articles were excluded because they were either published prior to the IADR meeting (*n* = 7), without access (*n* = 4), or were published in a non-English language (*n* = 3). The total of 114 remaining full-text publications were further analyzed for discrepancies with respect to their abstracts (Table 1). A total of 109 (95.6%) articles showed changes from their abstracts. Seventy four (65.0%) and 105 (92.0%) full-text publications had at least one major and one minor discrepancies, respectively (Table 2). The median number of major discrepancies between the abstracts and their full-text publications was 2.0 (range, 0–7), and the median number of minor differences was 2.0 (range, 0–5).

Overall, minor discrepancies were more prevalent (92.0%), compared to the major discrepancies (65.0%). The most common minor discrepancy was observed in the title (80.7%), followed by the changes in the last author (43.0%), number of authors (43.0%), first author (28.1%), and funding (21.9%) (Figure 2). 

Major discrepancies were commonly seen in the results section (48.2%) followed by changes in statistical analysis (43.9%), conclusion (38.6%), sample size (37.7%), materials and methods (33.3%), purpose of the study (31.6%), and study outcome (29.8%). Out of 43 articles that had discrepancy in the sample size, 30 articles had an increase in the sample size (70.0%), and 13 articles had a decrease in sample size (30.2%).

## 4. Discussion

IADR meetings offer a forum for the dissemination of dental research as well as education for many young researchers. Many of these abstracts are based on preliminary data and final conclusions, and implications are more appropriately made from a full-text publication [16]. Thus, it continues to be important for attendees to analyze and be aware of the quality of abstracts presented at these meetings. In addition, abstracts are read and used by many who do not attend the actual meetings [2, 29]. It is important for these individuals to be critical of the abstracts prior to publication as they were not present to hear the critiques provided by peers during the conference discussions.

Comparison between abstract and final full-text publication offers further insight into the quality of the abstracts presented at the IADR meeting, especially in Prosthodontic Section. In this study, 65.0% of the full-text publication had at least 1 major inconsistency, while 92.0% had at least 1 minor inconsistency, compared to their respective abstracts. Though minor inconsistencies may not be as critical in quality or validity of the research, such discrepancies should be kept to the minimum. 

In addition, low rates of major discrepancies like sample size (37.7%), materials and methods (33.3%), study objective (31.6%), and study outcome (29.8%) were found. These findings suggest high qualities of abstracts are presented at the Prosthodontic Section of the IADR meeting. It was, however, interesting to find that in 55 of the 114 manuscripts (48.2%), the results had changed which, in turn, changed the interpretation and conclusion of 44 manuscripts (38.6%). These occurrences, in addition to the rate of discrepancies, prompt attendees to be cautious. Bhandari et al. noted rates of discrepancies between the abstract and the full-text publication in terms of primary outcome measure and results, which differed 14% and 19% of the time, respectively [16]. In this study, the rates of discrepancies were higher than those of the Bhandari's with the highest amounts of major discrepancies of 48.2% in the results and 43.9% in statistical analysis. In addition, Corry reported a similar trend but a much lower prevalence of 30.2% authorship (name and number) and 69.8% title differences between randomly selected abstracts from the IADR/AADR and the corresponding published articles [21]. The substantial differences in authorship between the abstracts and the later publications in many disciplines may be attributed to the fact that abstracts are written for the poster presentation, which is often work in progress. Also, when authors submit their full-text articles as manuscripts for publication, their work is reviewed, and the authors make changes based on the feedback, which may lead to changes in the list of authors and their relative contributions to the final publication.

The reasons for the discrepancies in this study could be due to many factors. Some differences may exist to increase the chances of publication [2, 29]. The process of peer review during submission of a manuscript to a journal often leads to changes. This is due to incorporating the suggestions from the reviewers for the improvement of the manuscript [2]. Authors often omit giving details in the abstract due to the limitations in the number of words in the abstract submissions [2, 16]. It is difficult to include all the important information of methods, results, and discussion without crossing the critical word limit requirement of the meeting. Such information, however, is critical in understanding the abstract and should be considered as an important requirement for abstract submission at conferences. Standardization of abstracts and a thorough selection criterion for submission of abstract for conferences may minimize such discrepancies. During the process of full-text publication, all the important information which was otherwise omitted from the abstract are included. Therefore, readers are encouraged to search for the full-text publication in order to completely understand the content of the abstract [2]. 

 Although the abstracts submitted to the IADR meeting are peer reviewed, the feedback from the reviewers is usually not available to the abstract presenters, limiting their chance of modification and, in turn, limiting their interpretation. Furthermore, the requirement for abstract submission is often simpler and concise than those for full-text submissions. This does not reflect on the quality of the abstract, as they serve a different purpose and that is to encourage more abstract submissions at the meeting to maximize the exchanges of novel ideas and research information. 

Another reason for discrepancies may be because abstracts are often based on preliminary data [6, 12], and after additional data collection, sample size, results, and conclusions are modified. In general, changing sample size can be problematic particularly when there is a reduction in sample size from the abstract to full-text publication without explanation as to why the data were dropped from the study. Subjects lost to followup or excluded after adjusting methodology should be reported in the full-text publication, especially when included in the presented conference abstract [2]. This would assist the readers to determine the quality of the study. 

 An increase in sample size in full-text publication seems logical in order to increase the power of the analysis, especially in situations where data presented in the abstract is preliminary. If the additional data changes the author's conclusions, the meeting attendee is misinformed because he/she has only taken into consideration the information from the abstract and not from the final full-text publication. In the present study, 43 full-text articles had a change in sample size. Out of these 43, 30 articles (70%) had an increase in sample size, and 13 (30.2%) had a decrease in sample size. A 70% increase in sample size is a high number and would result in attendees getting an inaccurate picture of the study. Due to the change of the sample size, the final conclusion changed in 38.6% of the full-text publications. This study did not examine in detail the causes for changing the sample size variable and, therefore, cannot draw further inferences. 

Another possible reason that abstracts are being modified after presentation relates to the process of presenting data at national meetings. Criticisms during the meeting may lead to such modifications. The critical discussion following the presentation puts the study into better perspective than just reading the abstract without attending.

IADR has established structured abstract requirement for abstract submission process such as objective, methods, result, conclusion, and funding. Establishing a thorough selection criterion and implementation of a structured abstract format for submission is recommended to improve the quality of abstracts and minimize the discrepancies between the abstracts and their full-text publications [1, 3, 15, 29, 30]. The use of structured abstract was proposed by Haynes et al. [31] and consists of the following headings: objective, design, setting, patients, intervention, main outcome measures, results, and conclusions. Incorporating this format may also facilitate the review process for both the reviewer and the author. 

There are several potential limitations to our study. The years 2004 to 2005 were selected, so that it could represent the latest cross-section of abstracts in the prosthodontic research, assuming that most abstracts are followed by a full-text publication within 5 years [17, 19]. It is possible that some abstracts were published after the literature search was performed, or that some have yet to be published. In addition, few full-text publications that are not indexed in the PubMed database might have been omitted in the analysis. It is also possible that some abstracts may be presented after their full-text manuscript was published. In this study, such full-text publications were not included in the analysis because focus was on the change from abstract to publication. While this study presents descriptive data, the study does not analyze each abstract for the reasons for discrepancy. A systematic survey to analyze why these differences occur could offer a valuable insight into discrepancies observed in full-text publications.

## 5. Conclusions

Within the limitations of this study, the following conclusions were drawn.

1. A large number of discrepancies (*N* = 109, 95.6%) from the abstract to the full-text publication were observed. Minor discrepancies were more prevalent (92%) when compared to the major discrepancies (65%).
2. The data presented in this study may be useful in the development of abstract inclusion and grading criteria for future meetings and encourage the scientific committees to require higher standards for the abstract presentation.

## Figures and Tables

**Table 1:** Discrepancies between abstracts and their full-text publications.

Variables	*N*	Proportion
Overall	114	
No change	5	4.4%
Any change	109	95.6%

Minor discrepancy		

Number of authors		
No change	65	57.0%
Change	49	43.0%
(i) Increase	21	42.9%
(ii) Decrease	28	57.1%
First author		
No change	82	71.9%
Change	32	28.1%
Last author		
No change	65	57.0%
Change	49	43.0%
Title		
No change	22	19.3%
Change	92	80.7%
Funding		
No change	89	78.1%
Change	25	21.9%

Major discrepancy		

Statistical analysis		
No change	64	56.1%
Change	50	43.9%
Result		
No change	59	51.8%
Change	55	48.2%
Conclusion		
No change	70	61.4%
Change	44	38.6%
Sample size		
No change	71	62.3%
Change	43	37.7%
(i) Increase	30	70.0%
(ii) Decrease	13	30.2%
Purpose/objective		
No change	78	68.4%
Change	36	31.6%
Materials and methods		
No change	76	66.7%
Change	38	33.3%
Study outcome		
No change	80	70.2%
Change	34	29.8%

**Table 2:** Percentages of major and minor discrepancies found in full-text publications compared with their respective abstracts presented at IADR annual meetings.

Variable differences	Major *N* (%)	Minor *N* (%)	Cumulative differences	Major *N* (%)	Minor *N* (%)
0	40 (35)	9 (8)		40 (35)	9 (8)
1	13 (11)	27 (24)	≥1	74 (65)	105 (92)
2	9 (8)	34 (30)	≥2	61 (54)	78 (68)
3	6 (5)	29 (25)	≥3	52 (46)	44 (38)
4	12 (11)	9 (8)	≥4	46 (41)	15 (13)
5	13 (11)	6 (5)	≥5	34 (30)	
6	9 (8)			21 (19)	
7	12 (11)				
